# Health Tract, No. 173

**Published:** 1863-09

**Authors:** 


HEALTH TRACT, No. 173.

### INSANITY.

INSANITY, lunacy, and madness are the same in nature, but different in degree; all mean excessive mental action. Imbecility and idiocy imply a want of mental energy. The latter is a deficiency of brain power; the former an excess. "Insanity" is a Latin word, and may include all the above, for it means simply "without health," as to the brain. The most common cause of insanity, in its usual acceptation, is the mind's dwelling too much on one or a few things, as witness inventors, great geniuses, and others. Very many in lunatic asylums are classed among those who have had some great trouble; disappointed affection; loss of a dear relative or bosom friend. Had any one of these been called to endure half a dozen other troubles, each of which was equal to the first, there would have been no derangement at all; simply because the nervous power would have been diverted into different channels, would have been apportioned off to different parts of the brain, and thus have divided the intensity of the action to several, instead of one. Any muscle of the body unused, shrivels in size and loses its power; that same muscle is increased in size and power, in proportion as it is largely used. It is a common observation that he who thinks and talks incessantly of one thing, is soon set down by his neighbors as "crazy on that subject," although sensible and clever on others. The practical inference is, divert the mind in all troubles; do not brood over misfortunes; don't cherish sad or melancholy meditations; don't gloat over gold; never allow your reflections to become inseparable from any one idea. When you begin to complain that you "can't sleep," from the mind's running on one particular subject, you are rapidly preparing yourself for the mad-house! The fear of poverty has made many a rich man go crazy; but mind, it was the man who had felt its pinchings in younger years. The hardest worked slave rarely goes crazy, because he has no abiding sorrow; no concern about to-morrow's bread; his labor in the day is mechanical, and the moment it is over he feels free; his mind dismisses all thoughts of work, runs home and revels in his supper and other animal instincts; infinitely freer from any corroding care than his master. In educated and elevated New-England, there are nearly ten times as many crazy people as in an equal number of South-Carolina slaves. Taking planters and slaves together, there are three times fewer insane persons in the South than in an equal number of New-Englanders. More crazy people come from the farm than from the city. There are not half as many deranged persons in five thousand inhabitants of the Western States, as in as many from glorious New-England. One general principle explains these apparent contradictions. New-England is thickly settled; its soil is sterile, and the competition for bread is ceaseless and terrific. During its long and comparatively inactive winters, the mind frets at doing nothing; it is like a caged lion; it beats unavailingly against its prison-bars, and wastes itself in castle-building; in "vain thoughts!" To be without money is to be without bread in New-England; in the sunny South, and fruitful, blooming West, people "take trust for pay" literally; and can live for years on confidence and credit, and so in the South; in both sections pay-day is indefinitely postponed. Ohio is a fertile State, but thickly settled; these two things antagonize each other; hence the number of insane is half way between New-England and the South and West. In proportion as one idea, good or bad, absorbs the mind, in the same proportion is insanity courted.

